# Employee green behavior: Bibliometric-content analysis

**DOI:** 10.1016/j.heliyon.2024.e31045

**Published:** 2024-05-10

**Authors:** Xinxin Zhang, Siti Aisyah Panatik, Na Zhang

**Affiliations:** a School of Human Resources Development & Psychology, Universiti Teknologi Malaysia, Johor Bahru, Johor, Malaysia; b School of Foreign Languages for International Business, Hebei Finance University, Baoding, Hebei, China; c School of Economics and Management, Beijing Information Science & Technology University, Beijing, China

**Keywords:** Employee green behavior, Bibliometric analysis, Content analysis, Organizational sustainability, Web of science

## Abstract

**Purpose:**

– This research conducted a thorough literature review to analyze current research on Employee Green Behavior (EGB) and its contexts. It identified key areas of emphasis in EGB and provided a precise roadmap for future research.

**Design/methodology/approach:**

The research on employee green behavior will be visually represented using bibliometric analysis. Additionally, we provided a comprehensive overview of the correlation between relevant theories and determinants of employee green behavior. We also offered specific suggestions for future research to deepen consumer researchers' understanding of this concept.

**Findings:**

A bibliometric analysis was conducted on 275 journal articles regarding employee green behavior sourced from the Web of Science database spanning 2012 to 2023. The study revealed an increasing focus on employee green behavior research across diverse fields such as management, economics, psychology, sociology, and law. China, the United States, and Italy emerged as the top three countries both in publication volume and literature centrality. The academic literature primarily centers on investigating antecedent variables of green employee behavior, broadly categorized into corporate social responsibility, organizational support, green human resource management, transformational and ethical leadership, organizational commitment, job satisfaction, responsible leadership, intentions, values, organizational identification, and organizational climate. To comprehensively analyze prior studies, content analysis was performed, outlining and scrutinizing four empirical areas including antecedents, mediators, moderators, and outcomes of employee green behavior. While research on antecedents and influencing mechanisms of green loyalty was prevalent among the influencing variables, boundary conditions such as moderators received relatively less attention.

**Originality/value:**

This comprehensive literature review offers a clear understanding of the conceptual content, organization, and assessment of employee green behavior. Furthermore, it includes a bibliometric analysis of 275 papers on employee green behavior published between 2012 and 2023 in the Web of Science database. The text analysis reveals insights into the theoretical foundation, antecedent and outcome variables, and processes of employee green behavior, providing valuable guidance for future research.

## 1 Introduction

Organizations are compelled to diminish their environmental impact for enhanced environmental effectiveness, given recent organizational concerns about the implications of operations and programs on diverse environmental complexities, prompted by high stakeholder demands or methods for achieving comparative advantage [1]. Consequently, organizations should not solely prioritize financial aspects but also endeavor towards business sustainability (BS) [2]. Employee green behavior (EGB) emerges as a proactive approach to bolster environmental sustainability and mitigate issues, garnering corporate attention [3].

Studies on employee green behavior have broadened the focus of sustainability management from the organizational to the individual level, acknowledging the significant contribution that employees make to sustainability programs [4]. Subsequent research has explored various applications of employee green initiatives [5,6], examined factors influencing their frequency [7,8], and demonstrated their broader impact on overall organizational outcomes beyond the environmental sphere [9]. Additionally, these studies have deepened our understanding of the barriers hindering the acceptance of eco-friendly practices among employees [6] and the relationship between these behaviors and various performance standards [10]. Over the past two decades, research on green behavior among employees has gained increasing recognition. Numerous interdisciplinary fields, including engineering, tourism, ecology, management, and psychology, have investigated employee green behavior. However, the management literature on this topic is fragmented and primarily focuses on contextual factors, drivers, and theories, requiring further understanding. The researchers in this study aimed to examine the characteristics, evolution, and research trends of employee green behavior literature, with a specific emphasis on future research directions, given its multi-perspective and multi-oriented nature.

It should be noted that there is limited bibliometric research on environmental management, with existing studies primarily focusing on household pro-environmental behavior [[11], [12], [13]]. Recently, Phulwani conducted citation and bibliometric analyses on 788 publications to examine recycling behaviors among customers or households using VOSviewer and Gephi software [12]. Similarly, Lu utilized citation, co-word, and cluster analyses to systematically evaluate the evolution of pro-environmental behavior research, highlighting trends and advancements since the 1970s [13].

To gain a thorough understanding of the concept, it is imperative to conduct a comprehensive examination of employee green behavior. This article undertakes a bibliometric-content analysis to determine the intellectual organization and publishing output within this domain. Moreover, the study enhances our understanding by analyzing the research domain's structure from both conventional and temporal perspectives. Consequently, this study addresses the following research questions.

RQ1 What is the present understanding of the academic literature in the employee green behavior domain?

RQ2 What are the most prominent topics and themes of employee green behavior?

RQ3 What theoretical lenses have been employed by researchers to analyze employee green behavior?

RQ4 What are the variables in existing empirical research results and their relationships with employee green behavior?

RQ5 What are the future directions for employee green behavior research?

This review aims to make two contributions to the field of research on employee green behavior. Its primary objective is to identify areas of study receiving the most attention and to discern emerging patterns and tendencies related to employee green behavior. Utilizing bibliometric research techniques, we visually represent the research focus on employee green behavior. Furthermore, we offer a comprehensive overview of the correlation between relevant theories and determinants of employee green behavior, along with specific suggestions for future research to enhance understanding in this area for consumer researchers. This study seeks to consolidate and integrate existing studies and provides valuable suggestions for organizations to formulate effective strategies by systematically identifying factors influencing employee green behavior, thereby indicating potential avenues for future research.

The paper is structured as follows: initially, we explore the precise meaning and measurement of employee green behavior. Additionally, we employ bibliometric analysis to identify the focal points and trends of employee green behavior, providing a comprehensive overview of the field. This facilitates an impartial analysis of the extensive body of research on environmentally friendly employee behavior. Furthermore, content analysis is utilized to discuss the concepts employed in studying employee green behavior. Drawing upon previous research findings, we present a comprehensive theoretical framework elucidating employees' behavior concerning environmental sustainability. Finally, we offer numerous suggestions for further research to conclude the review.

## 2 Background

### 2.1 Conceptualization of employee green behavior

Initially, employee green behavior was viewed as an extension of pro-environmental behavior and a distinct form of organizational citizenship behavior [14]. During this period, the focus was primarily on the voluntary environmentally friendly actions of employees. However, through further exploration and considering oversight and regulatory aspects within businesses, the concept of employee green behavior evolved to include both voluntary environmentally friendly actions and job-related responsibilities. In other words, corporations now require green behavior as part of employees' duties. Consequently, these two environmentally friendly actions are increasingly integrated [15].

In the early phase of researching employee green behavior, it is often regarded as a distinct form of organizational citizenship behavior [16,17]. The definition and classification of employee green behavior are derived from the concept of organizational citizenship behavior. Initially introduced by Daily, the concept of OCBE refers to voluntary acts undertaken by employees within the organization that are neither incentivized nor obligatory, but aimed at improving the environment [18]. Unlike OCB, OCBE emphasizes individual behaviors aimed at minimizing personal and organizational resource usage and promoting sustainable growth within the company [19]. However, prior research has shown that not all employee green behaviors in the workplace are performed voluntarily by employees. Job responsibilities also influence employee green behaviors, contributing to the achievement of core business objectives [14]. Consequently, the concept of employee green behavior expanded beyond voluntary actions, leading to the establishment of the notion of employee green behavior.

Ones and Dilchert introduced the concept of employee green behavior (EGB), defined as “measurable actions and behaviors that employees undertake that either support or hinder environmental sustainability” [15]. The importance of this concept for advancing environmental sustainability within organizations is underscored by Andersson et al. (2013). According to Ones and Dilchert, employee green behavior encompasses both task performance and organizational citizenship behavior [15]. This assertion is corroborated by prior research conducted by Rotundo and Viswesvaran [20,21].

Employee green behavior stems from pro-environmental conduct [21], yet a fundamental distinction exists. While pro-environmental behavior encompasses all actions contributing to environmental well-being, both personally and professionally, employee green behavior specifically targets pro-environmental conduct within the workplace. Pro-environmental behavior is characterized by an individual's conscious and voluntary actions, evident across various aspects of life. This may involve purchasing eco-friendly vehicles, using organic cleaning products, reducing household electricity consumption, opting for public transportation, or adopting habits like limiting air conditioning use on hot days, relying on natural lighting, and minimizing shower duration. Employee green behavior necessitates organizational oversight, particularly in the absence of formal structures or job duties. It encompasses both voluntary and proactive environmental actions, as well as mandated environmental behaviors that employees must adhere to Ref. [10].

In summary, in conjunction with Ones and Dilchert's definition, this study posits that employee green behavior entails measurable pro-social and moral conduct exhibited by employees in the workplace or in tasks contributing to environmental sustainability [15].

### 2.2 Measurement of employee green behavior

Scholars have developed various categorization methods to capture and evaluate different types of green behaviors. The categorization of employee green behavior largely consists of three perspectives. The first perspective, centered on organizational citizenship behavior, defines green behavior as voluntary actions undertaken by individual employees. The second perspective regards employee green behavior as a unified concept. The third perspective considers organizational attributes, including supervision, and suggests that employee green behavior includes both in-role and extra-role behaviors. The detailed scale is provided in Table 1.

a. From the perspective of OCBE

**Table 1:** Representative measurement scale for employee green behavior.

Constructive Features	Author	Dimensions
Viewing employee green behavior as voluntary behavior	Wang X, Zhou K, Liu W (2018) Robertson J L, Barling J (2013)	1. I print double side whenever possible. 2. I put compostable items in the compost bin 3. I put recyclable material (e.g. cans, paper, bottles, batteries) in the recycling bins. 4. I bring reusable eating utensils to work (e.g. travel coffee mug, water bottle, reusable containers, reusable cutlery). 5. I turn lights off when not in use. 6. I take part in environmentally friendly programs (e.g. bike/walk to work day, bring your own local lunch day) 7. I make suggestions about environmentally friendly practices to managers and/or environmental committees in an effort to increase my organizations' environmental performance.
	Kim, Kim and Han (2014)	1. This group member avoids unnecessary printing to save paper. 2. This group member uses her or his own cup instead of a paper cup. 3. This group member uses stairs instead of elevators as much as she or he can when he or she moves to work in another office. 4. This group member reuses discarded papers to write memo or notes and messages when he or she works in the office. 5. This group member recycles reusable things in the workplace. 6. This group member resegregates reusable things that other team members do not recycle in the right recycle bins.
	Kaiser et al. (2017) Robertson J L, Barling J (2013) Kim et al. (2016) Safari. A. Salehzadeh. R., Panahi. R&Abolghasemian. S (2018)	1. In an effort to increase my organizations' environmental performance, I make suggestions and bring new ideas about environmentally friendly practices to environmental committees. 2. At work, I take part in environmentally friendly programs. 3. I share my knowledge about the environment with co-workers. 4. At work, I question practices that are likely to hurt the environment. 5. At work, I perform environmental tasks that are not required by my company.
	Baojie Zhang, Lifeng Yang, Xiangyang Cheng and Feiyu Chen (2021)	Four sub-dimensions Green learning 1. I stay informed of my company's environmental initiatives. 2. I actively participate in environmental protection related training provided by the company. 3. I take the initiative to learn environmental protection knowledge to improve environmental protection capabilities. 4. I print double-sided whenever possible. 5. I use personal water cups instead of disposable paper cups in the office. 6. I complete the tasks assigned by the company in an environmentally friendly way. 7. I perform the duties specified in the job description in an environment into account in everything they do at work. Influencing Others 8. I spontaneously give my time to help my colleagues take the environment into account in everything they do at work. 9. I encourage my colleagues to adopt more environmentally conscious behavior. 10. I encourage my colleagues to express their ideas and opinions on environmental issues. Organizational Voices 11. I make suggestions about environmentally friendly practices to managers to increase company's environmental performance. 12. I tray to draw management's attention to potentially environmentally unfriendly activities. 13. I inform management of potentially environmentally irresponsible policies and practices.
Dividing employee green behavior into voluntary employee green behavior (extra-role) and required employee green behavior (in-role)	Norton T A (2015) Bissing-Olson M J (2013)	Required employee green behavior 14. I adequately completed assigned duties in environmentally-friendly ways. 15. I fulfilled responsibilities specified in my job description in environmentally-friendly ways. 16. I performed tasks that are expected of me in environmentally-friendly ways. Voluntary employee green behavior 17. I took a chance to get actively involved in environmental protection at work. 18. I took initiative to act environmentally-friendly ways at work. 19. I did more for the environment at work than I was expected to.

The concept and classification of employee green behavior are informed by the connotation and categorization of organizational citizenship behavior. Boiral compared the six dimensions of organizational citizenship behavior, specifically addressing environmental organizational citizenship behavior: Helping behavior involves encouraging colleagues to act for the environment, aiding in environmental problem-solving, and collaborating across departments. Sportsmanship entails maintaining a positive attitude toward challenging environmental protection tasks that require extra effort. Organizational loyalty entails adherence to environmentally beneficial policies and goals, as well as participation in environmental activities on behalf of the company. Organizational compliance involves following the environmental practices set forth by the organization. Individual initiative encompasses actively engaging in workplace environmental activities, sharing pollution prevention knowledge, initiating new environmental projects, and openly discussing potential environmental activities. Self-development involves acquiring environmental knowledge, skills, and information through various channels to better understand and integrate environmental issues, participating in sustainable development training programs, and obtaining environmentally relevant information and technologies beneficial to the organization [22]. Paille and Boiral further delineated environmental organizational citizenship behavior into three dimensions: Eco-Initiatives encompass actions and proposals to enhance environmental practices and performance based on individual initiative behaviors, including workplace environmental protection actions, pro-environmental suggestions, and voluntary efforts to reduce greenhouse gas emissions. Eco-Civic Engagement involves voluntarily participating in organizational environmental activities to support and uphold the company's environmental initiatives, such as engaging in company environmental activities and promoting the organization's green image. Eco-Helping entails voluntarily assisting colleagues in integrating environmental issues into the workplace, offering support and assistance on environmental matters, encouraging colleagues to consider environmental issues, and promoting more environmentally responsible behavior among colleagues [23].

b. Specific behavior description

Ones and Dilchert delineated employee green behavior into five dimensions: Transformation involves integrating green concepts into product production and adopting sustainable innovation behavior; Resource Conservation entails reducing usage and practicing recycling, reuse, and recycling; Influencing Others includes encouraging and supporting others, as well as educating and training on sustainability; Taking Initiative encompasses prioritizing environmental concerns, developing programs and policies, and engaging in advocacy and action; Avoiding Harm entails preventing pollution, monitoring environmental impacts, and enhancing ecosystems [24].

c. In-role and extra-role behavior

Previous studies in the work-family domain suggest that individuals typically engage in green behaviors voluntarily; however, organizational psychologists argue that not all employee green behaviors are entirely voluntary. Norton and Parker categorized employee green behavior into two types based on task and relational performance: Required EGB and Voluntary EGB [10]. The aim is to assess, comprehend, elucidate, predict, and influence employee behavior concerning environmental sustainability at work. Required EGB pertains to green behaviors mandated by job roles or managerial directives, such as adhering to environmental policies, opting for eco-friendly alternatives, and adjusting traditional work practices, akin to task performance or indirect service related to the organization's core operations [10]. Voluntary employee green behavior refers to environmentally friendly actions initiated by employees without organizational directives, not constrained by rules or regulations. These actions, akin to relational (peripheral) performance and organizational citizenship behavior, involve adopting environmentally friendly work practices [8].

## 3 Methodology

### 3.1 Data collection

The Web of Science database was chosen for bibliometric analysis due to its extensive publication history and the inclusion of high-quality journals. The search terms encompassed “voluntary environmental behavior,” “employee green behavior,” “employee pro-environmental behavior,” “green behavior at workplace,” and “pro-environmental behavior at workplace” [25]. Articles like book reviews and editorial letters were excluded to ensure data quality. A total of 275 articles, spanning from 2012 to 2023, were selected for analysis.

### 3.2 Analysis method

#### 3.2.1 Bibliographic analysis

In recent years, there has been a growing interest among scholars in bibliometric analysis due to the widespread availability of published literature in easily accessible online databases [26]. Bibliometrics, an interdisciplinary field merging bibliography, informatics, mathematics, and statistics, facilitates quantitative analysis of existing literature. By employing scientific statistical methods, researchers can gain a comprehensive understanding of the historical and current trends in a field's development [27]. The utilization of bibliometric methodologies on a substantial collection of reference material offers a more comprehensive insight into a specific field compared to traditional literature surveys. Donthu categorize bibliometric analysis approaches into two groups: performance analysis and scientific mapping. Our objective is to analyze the general trends and focal areas related to environmentally conscious behavior among employees [28]. In line with Donthu et al.'s classification, bibliometric analysis methodologies are divided into scientific mapping and performance analysis [28]. We aim to identify broad study trends and hotspots concerning environmentally conscious workforce behavior. Hence, the bibliometric evaluation in this study adopts a scientific mapping approach to examine the interconnections among research components. Following Donthu recommendations, our study adheres to a four-step protocol for bibliometric analysis, involving defining the investigation's objectives and parameters, selecting analysis methods, collecting data, conducting the analysis, and summarizing the findings [28].

Step 1 This study aims to elucidate the bibliometric and intellectual structure of the existing literature on employee green behavior, specifically by determining the study's objectives and scope. The intellectual structure pertains to the primary subjects and themes of research, while the bibliometric structure reflects the publication output.

Step 2 This study employs a combination of bibliometric and content analysis tools to trace the evolution of literature on employee green behavior: Selection of Analysis Tools.

Step 3 Collect Data for Analysis: To retrieve articles and associated bibliometric and bibliographic data for analysis, refer to the instructions provided in the Data Gathering section of the preceding segment.

Step 4 Conduct the analysis and present the findings, which involves conducting bibliometric-content analysis and summarizing the results. To achieve this, bibliometric analysis is conducted using CiteSpace, and the results are visualized in a network [29],. CiteSpace is a visual tool for literature analysis that utilizes routing network techniques and co-citation analysis theories to depict the evolutionary trajectory, current state of research development, hot topics, and frontier areas within the selected research domain through the creation of various types of knowledge maps [30].

#### 3.2.2 Content analysis

This study also employs content analysis, a systematic method of coding and categorization to examine large amounts of textual data [31]. This method complements the bibliometric analysis by integrating rigorous quantitative analysis with qualitative approaches that preserve their nuanced meanings [32]. While bibliometric analysis reveals research hotspots and trends, it does not uncover the internal links and influencing processes of interconnected variables. Therefore, to offer a comprehensive overview of the theories and correlations among variables in the study of employee green behavior, literature content analysis was conducted.

## 4 Review results

### 4.1 Bibliographic analysis result

**Fig. 1:**
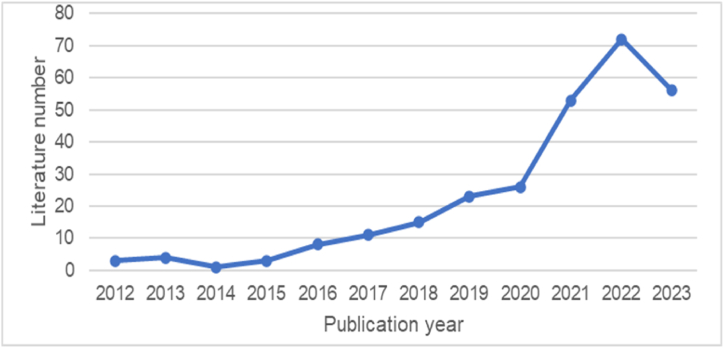
The number of documents from 2021 to 2023.

Fig. 1 depicts the document count for each year from 2012 to 2023, indicating a consistent upward trend in publications, particularly notable after 2020. The pinnacle of publication output occurred in 2022, with 72 articles. By mid-2023, 56 publications had already been recorded, suggesting sustained interest among researchers in environmentally friendly behavior among employees.

#### 4.1.1 Result of citation analysis

Various networks can be generated to depict the bibliographic landscape of green buildings, encompassing co-authors, co-cited documents, and keyword co-occurrence networks [33]. In this study, we concentrate on analyzing three primary dimensions: the document co-citation network, the clustering network, and the keyword co-occurrence network. Through document co-citation analysis, we pinpoint highly cited documents, serving as pivotal references in the field. Utilizing cluster statistics, we form clusters of closely linked documents based on their associations. To identify terms appearing in at least two distinct publications within a specific timeframe, the keyword co-occurrence network is utilized. Recognizing high-frequency and central keywords, as well as pivotal hotspots across time frames, contributes to building the knowledge base of green building. Employing CiteSpace aids in identifying significant references with notable citation bursts. Papers extensively cited during particular periods are considered milestones in the development of green building [30].

#### 4.1.2 Document Co-citation analysis

**Fig. 2:**
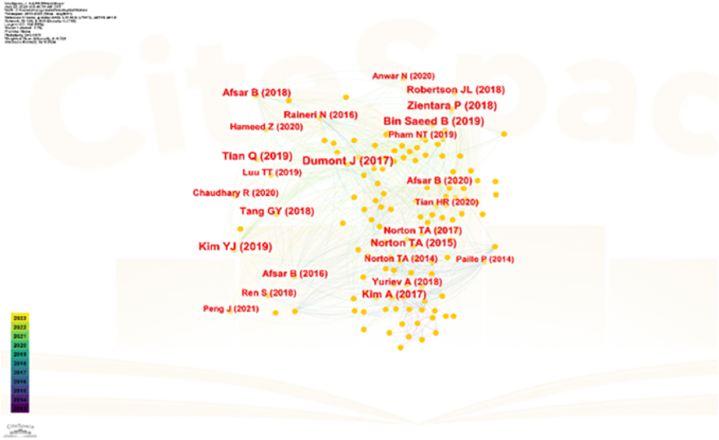
Document co-citation network of employee green behavior.

After visually inspecting 275 pages of the Web of Science database using CiteSpace, we select the top 50 publications with the highest citation frequency across all periods for co-citation analysis, constructing a co-citation network. From this network, we identify the ten most frequently co-cited papers and conduct further analysis. Fig. 2 presents these top 10 frequently co-cited papers from 2012 to 2023, while Table 2 provides additional details.

**Table 2:** Top 10 frequent co-cited documents in the employee green behavior.

Author	Title	Year	Freq.	Source
J Dumont, J Shen, X Deng	Effects of green HRM practices on employee workplace green behavior: The role of psychological green climate and employee green values	2017	73	Human resource management
Yong Joong Kim, Woo Gon Kim, Hyung-Min Choi, Kullada Phetvaroon	The Effect of green human resource management on hotel employees' eco-friendly Behavior and environmental performance	2019	60	International Journal of Hospitality Management
Bilal Bin Saeed, Bilal Afsar, Shakir Hafeez, Imran Khan, Muhammad Tahir, Muhammad Asim Afridi	Promoting employee's pro-environmental behavior through green human resource management practices	2019	54	Corporate Social Responsibility and Management
Piotr Zientara, Anna Zamojska	Green organizational climates and employee pro-environmental behavior in the hotel industry	2018	53	Journal of Sustainable Tourism
Tian, Q., & Robertson, J. L.	How and When Does Perceived CSR Affect Employees' Engagement in Voluntary Pro-environmental Behavior?	2019	51	Journal of Business Ethics
Andrea Kim, Youngsang Kim, Kyongji Han, Susan E. Jackson	Multilevel Influences on Voluntary Workplace Green Behavior: Individual Differences, Leader Behavior, and Coworker Advocacy	2017	48	Journal of Management
Bilal Afsar, Waheed Ali Umrani	Corporate social responsibility and pro‐environmental behavior at workplace: The role of moral reflectiveness, coworker advocacy, and environmental commitment	2019	44	Corporate Social Responsibility and Management
Jennifer L. Robertson and Erica Carleton	Uncovering How and When Environmental Leadership Affects Employees' Voluntary Pro-environmental Behavior	2018	36	Journal of Leadership & Organizational Studies
Bilal Afsar, Sadia Cheema, Farheen Javed	Activating employee's pro‐environmental behaviors: The role of CSR, organizational identification, and environmentally specific servant leadership	2018	35	Corporate Social Responsibility and Management
Guiyao Tang, Yang Chen, Yuan Jiang, Pascal Paille	Green human resource management practices: scale development and validity	2018	34	Asia Pacific Journal of Human Resources

Green Human Resource Management (HRM) serves as a significant driver for promoting employee green behavior. The first three cited documents support this assertion. Dumont elucidated the development of Green HRM as a concept aimed at influencing employee green behavior in the workplace [7]. Their findings revealed a direct impact of Green HRM on in-role green behavior and an indirect influence on extra-role green behavior. The psychological green atmosphere acts as a mediator in this indirect impact and extra-role green behavior. Kim demonstrated that Green HRM positively affects various organizational aspects, fostering increased organizational commitment among staff, promoting environmentally conscious conduct, and enhancing environmental performance in hotels [34]. Bin Saeed unveiled that Green HRM practices significantly enhance employees' pro-environmental behavior, with this relationship mediated by pro-environmental psychological capital [35]. Furthermore, the impact of Green HRM practices on pro-environmental behavior is moderated by employees' environmental knowledge. Additionally, Green performance management, green pay and rewards, green recruitment and selection, green training, and green engagement constitute the five pillars of Green HRM [36].

Scholarly interest in understanding the psychological mechanisms linking various antecedents to employee green behavior has intensified. Among the top 10 frequently co-cited documents, four delve into this particular aspect. Zientara and Zamojska reveal that Organizational Citizenship Behavior for the Environment (OCBE) is directly influenced by the Green Organizational Climate (GOC). Additionally, GOC significantly moderates the relationships between emotional organizational commitment and OCBE, and between personal environmental values and OCBE [37]. Unsworth suggest that employees' perceptions of corporate social responsibility indirectly affect their voluntary adoption of environmentally friendly practices, mediated by organizational identity [38]. Notably, these effects are more pronounced among employees with higher levels of empathy. Kim find that the voluntary workplace green activity of group leaders and individual group members correlates with conscientiousness and moral reflectiveness. The environmentally specialized transformational leadership impacts the voluntary pro-environmental conduct of employees both directly and indirectly [39].

Moreover, there is a claim that corporate social responsibility (CSR) serves as a crucial instrument for promoting effective social, environmental, and organizational performance. Tariq demonstrate that perceived corporate social responsibility influences pro-environmental behavior both directly and indirectly, with the indirect impact being mediated by organizational identification [40]. Afsar summarized that environmental commitment, colleague pro-environmental activism, and moral reflectiveness are all directly influenced by perceived corporate social responsibility [41]. Additionally, environmental commitment is significantly positively influenced by moral reflection and colleagues' pro-environmental activism. The association between perceived CSR and employee pro-environmental conduct is partially mediated by moral reflectiveness, environmental commitment, and colleague pro-environmental advocacy.

#### 4.1.3 Cluster identification and analysis

**Fig. 3:**
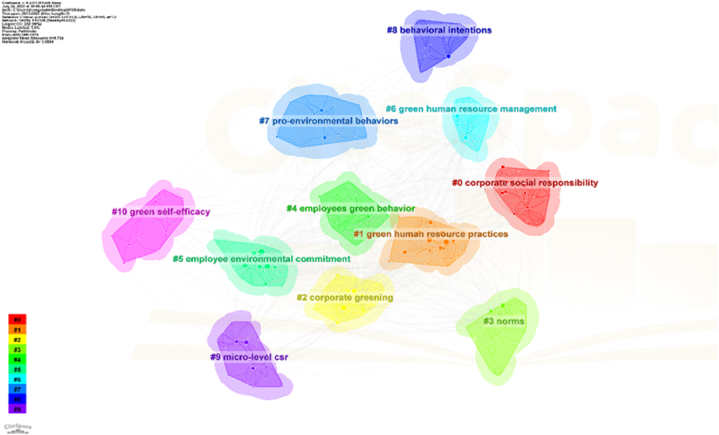
Clusters of knowledge domain within employee green behavior.

Initiating the construction of a knowledge domain begins by identifying frequently cited articles through document co-citation analysis. Subsequently, the materials are scrutinized to delineate the primary research area. Noun phrases from each cluster, derived from the document's title, keywords, and abstract, are utilized for selecting cluster labels, with priority given to the most frequently occurring phrases. The clusters generated by CiteSpace are depicted in Fig. 3, with Cluster 0 being the largest and Cluster 10 the smallest, based on the total number of publications within each cluster.

This finding underscores the current research emphasis on employee green behavior. Originating from investigations into organizational sustainable development and pro-environmental behavior [42], the focus has notably shifted towards understanding pro-environmental behavior, particularly in the workplace [[43], [44], [45]]. Scholars have predominantly explored the definition and dimensions of employee green behavior [46], followed by inquiries into the motivating factors behind such behavior, such as green human resource management practices [47]. Additionally, employee green behavior has garnered attention in interdisciplinary fields like environmental psychology [48], encompassing aspects such as employee environmental commitment, behavioral intention, and green self-efficacy [46,49].

Furthermore, as integral components of organizations, employee green behaviors serve as foundational elements for translating environmental management strategies into actionable practices [47]. Past research consistently highlights the significant role of corporate social responsibility (CSR) in shaping employee pro-environmental behavior (Mahmud [50,51]. The emerging concept of “micro-foundations of CSR,” focusing on individual actions and interactions, has gained traction and requires further exploration [52,53]. Investigating how employees' perceptions of CSR influence their engagement in pro-environmental behavior is crucial, given that employees are viewed as key stakeholders supporting organizational CSR initiatives and aligning their behavior with sustainable policies [54]. Consequently, several scholars have affirmed the interconnectedness between employee green behaviors and perceived corporate social responsibility (CSR) through various lenses [50,53,55].

#### 4.1.4 Keyword Co-occurrence network

**Fig. 4:**
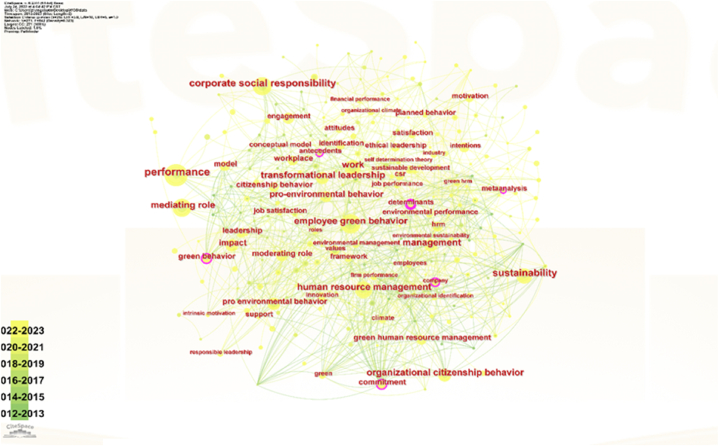
Keywords co-occurrence network.

The exploration of related keywords can elucidate the foundations of employee green behavior (EGB) due to the robust correlation between keywords and document cores. A keyword co-occurrence network is depicted in Fig. 4, with Table 3 showcasing the co-occurrence frequency, highlighting the most frequently used terms.

**Table 3:** Top-ranked clusters and the terms within the cluster.

No.	size	silhouette	mean（year）	label
0	28	0.715	2018	corporate social responsibility
1	27	0.659	2018	green human resource practice
2	27	0.783	2015	corporate greening
3	27	0.689	2017	norms
4	26	0.546	2021	employee green behavior
5	26	0.736	2017	employee environmental commitment
6	21	0.786	2015	green human resource management
7	17	0.74	2021	pro-environmental behaviors
8	16	0.843	2018	behavioral intentions
9	16	0.775	2019	micro-level CSR
10	14	0.724	2020	green self-efficacy

From Table 4, it is evident that the majority of the top twenty terms are predecessors of EGB. This suggests that earlier research has primarily focused on EGB's antecedents rather than its outcomes, including corporate social responsibility, organizational support, green human resource management, transformational leadership, ethical leadership, organizational commitment, job satisfaction, responsible leadership, intentions, values, organizational identification, and organizational climate. Similarly, a strong association between Organizational Citizenship Behavior (OCB) and leadership was observed. Ethical leadership (49 occurrences), responsible leadership (38 occurrences), and transformational leadership (55 occurrences) demonstrated the highest number of co-occurrences among various leadership styles in EGB research. Moreover, the study noted that the most prevalent and frequently referenced theories in the realm of EGB were the self-determination theory (29 occurrences) and the social planned theory.

**Table 4:** Top keywords with their frequencies in employee green behavior.

No.	keyword	freq.	No.	keyword	freq.
1	pro-environmental behavior	107	11	job satisfaction	44
2	sustainability	71	12	responsible leadership	38
3	corporate social responsibility/CSR	69/21	13	intentions	32
4	employee green behavior	62	14	values	32
5	organizational support	62	15	intrinsic motivation	31
6	green human resource management	61	16	organizational identification	31
7	transformational leadership	55	17	social planned theory	29
8	ethical leadership	49	18	organizational climate	28
9	organization commitment	47	19	Self-determination theory	28
10	organizational citizenship behavior	47	20	innovation	28

### 4.2 Theoretical foundations

#### 4.2.1 Theory of Planned Behavior (TPB)

According to this theory, research on employee green behavior can be divided into two primary categories: one primarily examines the correlation between intentions to engage in green behavior and the actual green behavior demonstrated by employees. For example, Norton suggested that the inclination to participate in environmentally friendly actions on one day influences the actual green behavior of employees on the following day, with the green psychological climate acting as a moderating factor [8]. To bolster the robustness of the findings, additional factors have been integrated into the Theory of Planned Behavior (TPB) framework, considering the specific context of various investigations. For instance, Lulfs and Hahn argue that the frequency and effectiveness of a company's previous initiatives can significantly impact its future operations [56]. They proposed incorporating habit as a mediator between behavioral goals and green behaviors. Blok explored the influence of values, personal norms, environmental awareness, and leadership support on behavioral intentions [42]. Carfora integrated pro-environmental self-identity and past actions into the TPB framework and found that self-identity plays a crucial role in influencing both past behaviors and behavioral intentions [57].

#### 4.2.2 Social norms theory

The social norms theory assesses the extent to which actions are socially acceptable and affects behavior by emphasizing the social consequences of participating (or not participating) in certain activities. This influence can be classified into descriptive norms, which depict commonly practiced behaviors, and prohibitive norms, which outline behaviors that are discouraged. de Araujo found that impression management influences employees' environmentally friendly behavior, with social norms serving as a moderating factor [58].

#### 4.2.3 Self-determination theory (SDT)

The self-determination theory suggests that individual behavior is shaped by a combination of autonomous and controlled motivation. Research on employee green behaviors is rooted in this concept, suggesting that such actions can be driven by voluntary, autonomous factors or by controlled factors imposed by corporate mandates. Norton and Parker introduced a research framework for employee green behaviors, emphasizing autonomous motivation and controlled motivation as the principal factors influencing such behaviors [59].

#### 4.2.4 Social exchange theory

The primary application of social exchange theory is to investigate the influence of businesses and colleagues on employees' green behavior. Paillé and Mejía-Morelos found that the perception of support from a company affects an employee's commitment to the organization, which in turn influences their environmentally friendly behavior [60].

### 4.3 Empirical research on employee green behavior

We provide a succinct overview of various types of factors (independent, mediating, moderating, and outcome variables) and their associations with employee green behavior. Furthermore, we propose an integrated model that encapsulates employee green behavior.

#### 4.3.1 Independent variables

The examination of independent variables influencing employee green behavior can be categorized into two groups based on the findings of the keyword co-occurrence network: situational factors and individual factors.

##### 4.3.1.1 Individual factors

There is a substantial body of research on individual-level employee green behavior, focusing primarily on personality traits, self-efficacy, environmental awareness, green values, and age. For instance, Kim investigated the influence of the personality trait of responsibility on employee green behavior [5]. They suggest that over time, employees may acquire the knowledge and skills necessary to engage in environmentally friendly activities, leading to an increase in their green self-efficacy - referring to their belief in their ability to undertake sustainable behaviors. Employees with such attitudes are more likely to actively adopt environmentally friendly practices and exert greater effort in sustainability-related tasks within the organization. Afsar argued that employees engage voluntarily in green behaviors only when they perceive these activities as congruent with their self-concept and group interests, suggesting that intrinsic motivation plays a pivotal role in driving green behaviors [41]. Wiernik explored the relationship between age and employee green behavior, finding that older employees demonstrate a greater inclination to participate in conservation activities and promote green behaviors among their colleagues. Additionally, they display a heightened commitment to mitigating environmental risks in the workplace and encouraging their peers to adopt environmentally friendly practices [61].

##### 4.3.1.2 Situational factor

There is a growing body of research examining how situational factors, such as corporate social responsibility, leadership behavior, green human resources management (HRM) practices, and Green Transformational Leadership, influence employee green behavior. Corporate Social Responsibility (CSR) holds significant importance for organizations as it shapes employees' perceptions of the firm, subsequently influencing their attitudes and behaviors toward it [62]. CSR encompasses the ethical and sustainable activities and policies employed by a corporation to enhance the welfare of its stakeholders [63]. The behavior of leaders plays a critical role in shaping employees' behavior. According to Robertson and Carleton, leaders communicate with employees by consistently exhibiting conduct that fosters a positive atmosphere [64]. This signaling cultivates an awareness among employees that such actions are highly valued and expected within the organization, prompting them to adopt and embody similar behavior, leading to favorable outcomes. Green HRM practices involve integrating environmental awareness into organizational management and utilizing human resource strategies to advance the organization's green strategic objectives, aiming to improve environmental outcomes and achieve sustainable development. Dumont found that implementing green HRM practices in an Australian multinational enterprise positively influences employee green behavior within their job responsibilities. Additionally, he highlighted the significant role of employees' personal environmental values in the relationship between green HRM practices and employee green behavior beyond their job responsibilities [7]. The study by Graves and Sarkis emphasized the importance of green transformational leadership, which emphasizes environmental objectives, in establishing a green vision for the organization [65]. This type of leadership motivates members to work toward green goals and encourages the adoption of environmentally friendly behaviors among employees, ultimately impacting employees' engagement in green behaviors positively.

#### 4.3.2 Mediating variables

The bulk of research on mediating variables has primarily focused on organizational support, emotional commitment, stakeholder values, job satisfaction, green motivation, green behavioral intentions, and organizational green climate. Paille and Raineri demonstrated that perceived organizational support can enhance individuals' job satisfaction, thereby promoting the development of environmentally friendly behaviors [48]. Norton found that corporate sustainability policies positively influence employees' green behaviors, particularly regarding mandatory versus voluntary actions. This effect is mediated by the perception of a green work environment [8]. Paille and Raineri's study underscored that employees' job satisfaction can drive their concern for the organization's sustainable growth and social responsibility, thereby fostering the adoption of environmentally friendly habits, commonly referred to as green behaviors [48]. Norton showed that a corporate green environment facilitates the adoption of task-oriented green behaviors by effectively communicating green goals and values to employees [66].

#### 4.3.3 Moderating variables

Only a limited amount of research has explored how employee green behavior, including positive emotion, empathy, green psychological climate, pro-environmental attitude, and social norms, may influence other factors. Ren demonstrated that intense positive emotions diminish the impact of stakeholder values on green activities [9]. Conversely, Tian and Suo found that empathy positively moderates the relationship between corporate social responsibility (CSR), corporate identity, and voluntary green activities [67]. Norton proposed that employees' green behavior on one day influences their green behavior on the next day, with the green psychological climate serving as a moderating factor [10]. Bissing-Olson et al. (2013) discovered that regular activation of positive emotions is associated with task-related environmentally friendly behavior among employees [14]. They also noted that pro-environmental attitudes significantly impact voluntary and task-related environmentally friendly behavior. Additionally, they observed that daily activation of positive emotions and pro-environmental attitudes act as moderators between daily activation of positive emotions and task-related environmentally friendly behavior among employees.

#### 4.3.4 Outcome variables

The impacts of employees' green behaviors are primarily evident in their job satisfaction, career development, and organizational green performance. Osbaldiston and Sheldon emphasized the role of goals in promoting and guiding green behavior patterns over time [68]. In a study, participants were asked to select three green goals achievable within a week, with a follow-up conducted after one week. Tracking revealed that employees' task-based green behaviors facilitated goal achievement, satisfying job requirements and leading to increased job satisfaction. Building on this, Bauer and Aiman-Smith explored the influence of employees' green orientation on career development. Their findings suggest that engaging in green behavior enhances individuals' social standing through their commitment to environmental protection, subsequently improving their professional image, job prospects, and promotion opportunities [69]. Additionally, Paille underscored the importance of employee support for implementing green policies and measures within organizations. They argue that employees' green citizenship behavior is instrumental in enhancing organizational green performance [70].

**Fig. 5:**
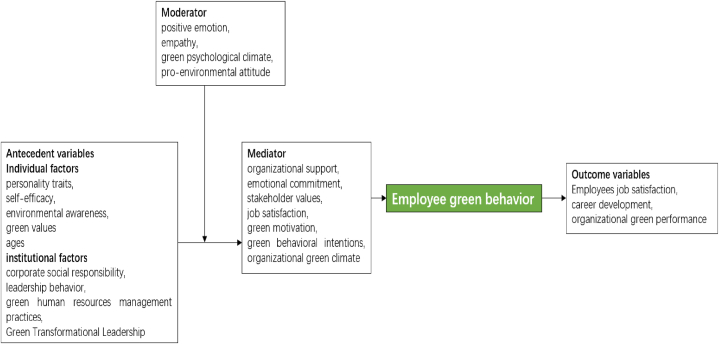
Integrated model of employee green behavior.

As seen in Fig. 5, we developed an integrated model based on the findings of the current empirical investigation.

### 4.4 Future research direction

Paul argued that systematic literature reviews should offer cutting-edge insights and thought-provoking objectives to advance knowledge in the reviewed field. In line with this perspective, we aim to address these research gaps and provide several recommendations for future investigation [71].

Individual traits within organizations shape employees' perceptions, thoughts, and ultimately influence their behavior [39]. Responsible individuals strive for optimal performance in their roles and consistently engage in activities that contribute to organizational growth. Kim examined the impact of the personality trait of responsibility on employees' green behavior [72]. However, their study only explored the influence of responsibility on environmentally-friendly behavior. Future research could explore the effects of additional personality traits, such as extraversion, on employee green behavior. Therefore, we propose the following recommendations.

Proposition 1 *Different types of personality traits* (*e*.*g*., *extraversion*) *will have independent impacts on employee green behavior*.

*Motivation serves as the intrinsic driving force behind employee behavior* [73]. *Kim investigated the influence of employee motivation*, *both autonomous and external*, *on green behavior*, *concluding that individuals driven by autonomous motivation are more likely to adopt green behaviors* [5]. *Such behaviors align with their intrinsic values and personal goals*, *providing them with a sense of fulfillment and satisfaction*. *Conversely*, *external motivation*, *manifested through rewards*, *praise*, *and other forms of organizational recognition*, *aims to incentivize employee behavior and reinforce work attitudes*. *Organizations employ environmental management systems and incentive programs to encourage green behaviors*, *thereby emphasizing their importance to employees*. *Afsar noted that employees will only engage in green behaviors when they perceive them as essential to their self-concept and collective interests*, *highlighting the role of internal motivation in fostering such behaviors* [41]. *However*, *this study solely focused on the impact of internal motivation on green behaviors*. *Future research could explore the effects of other forms of motivation*, *such as external and control motivation*, *on employees' green behaviors within this research framework*. *Therefore*, *we propose the following recommendations*.

Proposition 2 *external motivation will have independent impacts on employee green behavior*.

*There is a notable dearth of research concerning the weighting mechanism underlying employees' green behaviors*, *with many studies primarily focusing on emotions and cognition while neglecting cultural contexts*. *For instance*, *Dumont highlighted the moderating role of personal values in the relationship between green human resource management practices and extra-role green behaviors* [7]. *Mirahsaniet suggested that organizations can foster green behaviors by implementing policies related to green identity and creating supportive working conditions* [74]. *To address this gap*, *future research could delve deeper into the Chinese context*, *exploring the moderating role of factors with distinct local cultural characteristics*, *such as collectivism*. *Collectivism stands out as a prominent feature of Chinese cultural values and is extensively studied in the current Chinese context*. *In collectivist cultures*, *individuals perceive themselves as part of a group and prioritize interdependent relationships with other group members* [75]. *This collective mindset leads organizational employees to prioritize the broader interests and well-being of the group*. *Consequently*, *employees in such cultures are inclined to consider the collective welfare over personal gains or losses*, *thereby promoting the adoption of green behaviors and contributing to organizational sustainability*. *Future research can further validate the explanatory power of these insights*. *Thus*, *we propose the following recommendations*.

Proposition 3 *collectivism will play a moderator role between antecedent variables and employee green behavior*.

*Future research could delve into the impact of employee green behavior on the individuals themselves*. *Despite the considerable attention given to antecedent variables of employee green behavior*, *there remains a noticeable scarcity of research on its outcome variables*. *In recent years*, *scholars have increasingly emphasized the significance of pro-social behavior for individuals*. *Pro-social behavior*, *akin to "giving a rose and leaving a fragrance in the hand" from the perspective of the implementer*, *has been associated with a sense of efficacy and significance*, *contributing to individuals' well-being*. *Employee green behavior represents a specific manifestation of pro-social behavior*. *Consequently*, *when employees engage in green behaviors*, *they may experience positive changes*, *fulfilling their sense of efficacy and meaning*, *thereby enhancing their overall happiness*. *This suggests that employee green behaviors could potentially contribute to happiness*. *Building on this analysis*, *future research could delve deeper into this topic by examining happiness as an outcome variable*. *Thus*, *we propose the following avenues for future investigation*.

Proposition 4 *employee green behavior will have positive impact on employees’ happiness*.

## 5 Discussion

### 5.1 Theoretical implications

In addition to piquing the interest of numerous corporate managers, employee green behavior has also garnered significant attention from researchers. This review provides an impartial and comprehensive summary of the existing body of research on employee green behavior. Firstly, we offer a clear definition of employee green behavior and an overview of the concept along with its measurement techniques.

Secondly, we conducted a bibliometric analysis of 275 journal articles on employee green behavior sourced from the Web of Science database spanning from 2012 to 2023. The analysis revealed a growing interest in researching employees' green behavior across various disciplines, including management, economics, psychology, sociology, and law. The top three countries in terms of publication volume and centrality of the literature were identified as China, the United States, and Italy. Academic output predominantly focuses on antecedent variables of employee green behavior, encompassing areas such as corporate social responsibility, organizational support, green human resource management, transformational leadership, ethical leadership, organizational commitment, job satisfaction, responsible leadership, intentions, values, organizational identification, and organizational climate. However, these studies often address these variables unilaterally, lacking systematic considerations. Moreover, there is a notable dearth of research on outcome variables compared to antecedent variables. Concurrently, the existing research on green behavior exhibits several shortcomings that warrant attention.

Thirdly, to better leverage the content of previous studies, we conducted content analysis. We outlined and examined four areas of empirical study, including the theories employed in earlier research: antecedent, mediating, moderating, and outcome factors influencing employee green behavior. While research on the antecedents and influencing mechanisms of employee green behavior was relatively abundant, factors such as boundary conditions and moderators received less attention.

### 5.2 Practical contribution

First, enhancing research on antecedent variables of employee green behavior requires considering different socio-cultural contexts in various societies. While studies on antecedent variables are more prevalent than those on outcome variables, existing research often neglects regional and cultural disparities across different countries. Future studies should explore the influence of employee green behavior formation processes, taking into account cultural values. Additionally, employee green behavior should be perceived as the outcome of multiple factors influenced by leadership behaviors, individual values, and the organizational green climate, among other factors. Subsequent studies should comprehensively consider these influencing factors and analyze how these multilayered elements interact to promote the adoption of green behaviors by employees.

Second, there is a need to strengthen research on outcome variables related to employee green behavior, including their impacts on organizations, teams, and individuals. By investigating the effects of employee green behavior on organizations, teams, and individuals, scientific research can better encourage the organic adoption of green behaviors by organizations and employees. Compared to research on the influencing factors of employee green behavior, studies on its outcomes are not only limited but also scarce in empirical research. Future research can endeavor to conduct empirical studies to ascertain whether employee green behavior can further enhance collective environmental awareness, thereby promoting the adoption of group green behaviors.

Third, there is a need to develop a research scale for employee green behavior tailored to the Chinese context. Most existing scales for employee green behavior are based on cultural contexts outside of China and lack specificity for the Chinese context. Given China's significant emphasis on environmental issues, there is potential to develop a scale for employee green behavior with Chinese characteristics based on real-life situations in China. This initiative could advance research related to employee green behavior in China.

### 5.3 Limitations

This study has several limitations. Firstly, the knowledge mapping analysis function and bibliometric approaches of the software are constrained by the limited quantity and coverage of research article data available in the Web of Science database. Additionally, biases may arise during the data collection process, statistical analysis, and examination of study samples. To address these limitations, future research endeavors should aim to gather more comprehensive data on employee green behavior, employing a variety of qualitative and quantitative analytical techniques and tools to conduct a more thorough and unbiased analysis and comparative study of its progression. Despite the substantial volume of data analyzed in this study, the depth of analysis regarding factors associated with employee green behavior is insufficient. Further investigation is warranted to provide a more nuanced understanding of these factors.

## 6 Conclusions

The research focus on employee green behavior will be visually represented using bibliometric research techniques. Additionally, we provide a comprehensive overview of the precise correlation between relevant theories and determinants of employee green behavior, accompanied by specific suggestions for future research to enhance researchers' understanding of this concept. Future research could partially or fully implement conceptual and methodological frameworks based on these recommendations. Addressing current literature gaps, this study's agenda has the potential to guide and support advanced research for optimal environmental management and employee green behavior. These suggestions may also complement studies focusing on Employee Green Behavior (EGB). Implementing the recommendations could shed light on the attributes and limitations of successful EGB application processes. Moreover, the proposed future directions could offer valuable insights for conducting authentic studies and facilitating teaching processes and empirical examinations of increasingly popular topics such as environmental management, EGB, and Green Human Resource Management (GHRM) across diverse settings.

## Data availability statement

No data was used for the research described in the article.

## CRediT authorship contribution statement

**Xinxin Zhang:** Methodology, Investigation, Formal analysis, Conceptualization. **Siti Aisyah Panatik:** Project administration. **Na Zhang:** Writing – original draft, Supervision, Funding acquisition.

## Declaration of competing interest

The authors declare that they have no known competing financial interests or personal relationships that could have appeared to influence the work reported in this paper.
